# Oral supplementation of choline attenuates the development of alcohol-related liver disease (ALD)

**DOI:** 10.1186/s10020-024-00950-4

**Published:** 2024-10-18

**Authors:** Victor Sánchez, Anja Baumann, Franziska Kromm, Timur Yergaliyev, Annette Brandt, Julia Scholda, Florian Kopp, Amélia Camarinha-Silva, Ina Bergheim

**Affiliations:** 1 Department of Nutritional Sciences, Molecular Nutritional Science, University of Vienna, Josef-Holaubek-Platz 2 (UZA II), A-1090 Vienna, Austria; 2 Livestock Microbial Ecology Department, Institute of Animal Science, University of Hohenheim, Stuttgart, Germany; 3 Department of Pharmaceutical Sciences, Clinical Pharmacy Group, University of Vienna, Vienna, Austria

**Keywords:** Intestinal barrier, Ethanol, Nitrite, Lieber DeCarli diet, Choline oxidase

## Abstract

**Background:**

Chronic alcohol intake is associated with alterations of choline metabolism in various tissues. Here, we assessed if an oral choline supplementation attenuated the development of alcohol-related liver disease (ALD) in mice.

**Methods:**

Female C57BL/6 J mice (n = 8/group) were either pair-fed a liquid control diet, or a Lieber DeCarli liquid diet (5% ethanol) ± 2.7 g choline/kg diet for 29 days. Liver damage, markers of intestinal permeability and intestinal microbiota composition were determined. Moreover, the effects of choline on ethanol-induced intestinal permeability were assessed in an ex vivo model.

**Results:**

ALD development as determined by liver histology and assessing markers of inflammation (e.g., nitric oxide, interleukin 6 and 4-hydroxynonenal protein adducts) was attenuated by the supplementation of choline. Intestinal permeability in small intestine being significantly higher in ethanol-fed mice was at the level of controls in ethanol-fed mice receiving choline. In contrast, no effects of the choline supplementation were found on intestinal microbiota composition. Choline also significantly attenuated the ethanol-induced intestinal barrier dysfunction in small intestinal tissue ex vivo, an effect almost entirely abolished by the choline oxidase inhibitor dimbunol.

**Conclusion:**

Our results suggest that an oral choline supplementation attenuates the development of ALD in mice and is related to a protection from intestinal barrier dysfunction.

## Background

Despite a global decrease in alcohol consumption in recent years, in many European countries the alcohol intake remains high (Pruckner et al. 2019) ranging from 8 to 12 L of pure alcohol consumed per adult > 15 years of age each year (Pruckner et al. 2019). Chronic elevated alcohol intake is also still a major cause of liver-related mortality worldwide (Griswold et al. 2018; Asrani et al. 2019). Alcohol-related liver disease (ALD) comprises a broad spectrum of disorders ranging from simple hepatic steatosis to progressive injury including steatohepatitis, fibrosis, cirrhosis, and even hepatocellular carcinoma (Seitz et al. 2018). To date, abstinence is the therapy of choice to prevent the onset but also progression of ALD. However, especially in settings of alcohol dependence, relapse rates are high. Also, studies suggest that steatosis may increase the probability of developing later stages of disease e.g., fibrosis and cirrhosis (Day and James 1998; Harrison et al. 2002). Therefore, a better understanding of molecular mechanisms associated with the development of ALD may help to establish better strategies to attenuate the disease´s progression.

Results of many studies suggest that both acute and chronic consumption of large amounts of alcohol, e.g., > 20 g raw ethanol within 1–2 h (Bode et al. 1987; Bala et al. 2012), can cause intestinal barrier dysfunction going along with an increased permeation of bacterial endotoxin and so-called pathogen-associated molecular pattern (PAMPs) into the circulation e.g., the portal vein (for overview see (Hsu et al. 2023)). In the liver, these bacteria-derived compounds have been shown to bind to Toll-like receptors (TLR, especially TLR4) found on Kupffer cells but also other cells, thereby activating dependent signaling cascades and resulting in an enhanced formation of reactive oxygen species like nitric oxide (NO) and induction of the expression of proinflammatory cytokines including tumor necrosis factor alpha, interleukin (IL)-6 and IL-1β (for overview see (Tang et al. 2023)). Moreover, results of mainly pre-clinical but also some clinical studies have shown that targeting either intestinal microbiota, or barrier dysfunction as well as TLR signaling cascades, and herein, especially TLR4, may dampen the development of ALD (for overview see (Ranjbarian et al. 2023)).

While humans have been shown to be able to a de novo synthesis of choline (Zeisel and Costa 2009), nutrition societies account choline to the essential nutrients for humans (Arias et al. 2020). The recommended adequate intake for choline is ~ 550 mg/d, but as summarized by Mehedint et al., epidemiological studies suggest that the lowest and highest quartile of intake are ~ 150 mg and ~ 500 mg/d (Mehedint and Zeisel 2013). It has been shown that a diet low in choline is associated with the development of fatty liver in humans and can in some individuals even be associated with significant hepatic damage (Fischer et al. 2007; Guerrerio et al. 2012). In line with these findings, already more than 60 years ago, it has been proposed by the results of animal studies that liver damage associated with the intake of alcohol can be prevented by choline supplementation (Best et al. 1949) which later has been confirmed in humans with metabolic dysfunction-associated steatotic liver disease (Kansakar et al. 2023). Choline serves as a precursor for the synthesis of acetylcholine, betaine, phospholipids, and trimethylamine (for overview also see (Arias et al. 2020)). Studies in rodents suggest that in settings of a developing ALD the availability of both, betaine and phospholipids, may be critical (Arumugam et al. 2021) and that an oral supplementation of choline may attenuate or at least diminish the development of ALD (Arumugam et al. 2021). However, some older studies in baboons (Lieber et al. 1994) suggest that choline may also have adverse effects in settings of liver fibrosis when being supplemented at high concentrations (100 mg/1000 kcal). Also, molecular mechanisms underlying the protective effects of choline on the development of (alcohol-related) liver damage are not yet clarified.

Starting from this background, the present study aimed to determine if an oral supplementation of choline attenuates the development of alcohol-related liver damage in female C57BL/6 J mice and if so, to assess whether this is related to a protection from alcohol-induced intestinal barrier dysfunction. Furthermore, employing small intestinal everted tissue sacs molecular mechanisms associated with the effects of choline on ethanol-induced intestinal barrier dysfunction were determined ex vivo.

## Methods

### Animals and treatment

Animal feeding experiments were approved by the local Institutional Animal Care and Use Committee (IACUC; Austrian Federal Ministry of Education, Science and Research, 2022–0.039.040). Female animals were obtained from Janvier SAS, France and housed in a pathogen-free barrier facility accredited by the Association for Assessment and Accreditation of Laboratory Animal Care (AAALAC) in accordance with the Austrian animal welfare laws. After adapting to the animal facility, mice were randomly assigned to the following four feeding groups: mice fed a control Lieber DeCarli diet (C) or control Lieber DeCarli diet enriched with 2.7 g choline/kg diet (C + choline; Lieber DeCarli diet obtained from IPS Product Supplies Ltd, Alfreton, UK; choline obtained from Fisher Scientific, Vienna, Austria) or an ethanol containing Lieber DeCarli diet (EtOH, 5% ethanol; Sigma-Aldrich, Steinheim, Germany) or ethanol containing Lieber DeCarli diet enriched with 2.7 g choline/kg diet (EtOH + choline). The concentration of choline was based on previous studies of others (Jacobs et al. 2010). All mice were adapted to the intake of a control Lieber DeCarli liquid diet for 12 days. Mice included in the ethanol treatment group were then adapted to the intake of ethanol by gradually increasing the concentration from 2% ethanol by 1% every third day. Maximum ethanol concentration (5%) was fed for 20 days. Mice had ad libitum access to drinking water at all times. Consumption of diets was controlled and adapted daily, and body weight was assessed weekly. At the end of the feeding experiment, mice were anesthetized with a ketamine/xylazine mixture (i.p. injection, 100 mg ketamine/kg bw; 16 mg xylazine/kg bw) and killed by cervical dislocation. Blood was collected from the portal vein, and liver as well as intestinal tissue were collected and either fixed in neutral-buffered formalin or snap-frozen and stored at − 80 °C. Moreover, small intestinal tissue was collected to build everted tissue sacs to determine xylose permeability as detailed before (Rajcic et al. 2021) and described briefly below.

### Everted tissue sac model of mice ex vivo

Naïve female C57BL/6 J mice were killed by cervical dislocation. As detailed previously, small intestine was rapidly removed and everted with a rod (Rajcic et al. 2021). In brief, small intestinal tissue was cut into segments equal in length and put into cold DPBS. Each sac was filled with 1 × Krebs–Henseleit-bicarbonate-buffer containing 0.2% (w/v) bovine serum albumin (KRH buffer). Experiment 1: Sacs (n = 5–6) were incubated for 55 min at 37 °C in gassed KRH buffer (95% O_2_/5% CO_2_) ± 0.8 g ethanol/L. In addition, some tissue sacs were concomitantly incubated with 0.7 mM choline (choline chloride, Santa Cruz Biotechnology, Dallas, TX, USA) for 55 min. After 55 min 0.1% (w/v) D-xylose (Merck KGaA, Darmstadt, Germany) was added to the KRH buffer to assess permeability. Experiment 2: To determine if choline oxidase is critical in mediating the effects of choline on ethanol-induced intestinal barrier dysfunction sacs (n = 6–7) were incubated for 55 min at 37 °C in gassed KRH buffer (95% O_2_/5% CO_2_) ± 0.8 g ethanol/L ± 10 µM choline oxidase inhibitor (dimbunol; Sigma-Aldrich, Steinheim, Germany). The concentration of the inhibitor used was based on the results of previous studies of others (Barlow and Marchbanks 1985) and pilot experiments (data not shown). As xylose permeation was similar when comparing control sacs and those challenged with choline, or the inhibitor only results of control (C) are shown. In the case of the feeding experiment, everted sacs from the different treated mice were also incubated for 5 min in 0.1% (w/v) D-xylose. Intestinal tissue from each sac was snap frozen for further analyses.

### Xylose assay

A xylose assay, which is based on a reaction of xylose with phloroglucinol, was performed as detailed before (Eberts et al. 1979).

### Blood parameters of liver damage and histological evaluation of liver sections and inflammatory alterations

Alanine aminotransferase (ALT) activity in plasma of mice was measured in a routine laboratory (University of Veterinary Medicine, Vienna, Austria). Sections of paraffin-embedded livers (4 µm) were stained with hematoxylin and eosin (Sigma-Aldrich, Steinheim, Germany), and the Nanji score was used to evaluate liver histology (Nanji et al. 1989). To determine hepatic lipid accumulation, frozen liver sections (10 µm) were stained with Oil-Red O (Sigma-Aldrich, Steinheim, Germany) for 5 min and counterstained with hematoxylin for 20 s. Using a commercially available kit neutrophil granulocytes were stained in liver sections (Naphthol AS-D Chloroacetate Specific Esterase Kit; Sigma-Aldrich, Steinheim, Germany) as explained before (Spruss et al. 2012) or were stained with lymphocyte antigen 6 complex locus G6D (Ly6G) antibody (Abcam, Cambridge, UK) as detailed previously (Sanchez et al. 2024). The number of neutrophil granulocytes and Ly6G-positive cells per microscopic field (magnification 200 x, 8 fields/section) was assessed with a camera integrated in a microscope (Leica DM6 B, Leica, Wetzlar, Germany).

### Detection of nitrite (NO_x_) in liver and intestinal tissue

NO_x_ levels were assessed in small intestinal and liver tissue using a commercially available Griess reagent kit according to the manufacturer´s instructions (Promega, Madison, WI, USA) and as detailed previously (Rajcic et al. 2021).

### ELISA measurement and endotoxin assay

Using a commercially available ELISA kit (DuoSet ELISA Kits, R&D Systems, Minneapolis, MN, USA) protein concentration of IL-1β and IL-6 were determined in liver tissue according to the manufactures instruction. To determine bacterial endotoxin levels in portal plasma of mice a commercially available limulus amebocyte lysate assay (Charles River, Ecully, France) was used.

### Immunohistochemical staining of 4-hydroxynonenal (4-HNE) protein adducts and F4/80 in liver tissue as well as of zonula occludens 1 (ZO-1) in small intestinal tissue

Sections of paraffin-embedded liver tissue were cut (4 µm) and stained to determine the concentration of 4-HNE protein adducts as detailed before (Jin et al. 2015). In brief, after endogenous peroxidase blocking sections were incubated with a polyclonal antibody for 4-HNE (AG Scientific, San Diego, CA, USA) and then incubated with the respective secondary antibody and diaminobenzidine solution (Agilent, Santa Clara, CA, USA). Moreover, liver sections were stained for F4/80-positive cells (F4/80 antibody: Abcam Cambridge, UK) as described previously (Brandt et al. 2019). Sections of paraffin-embedded small intestinal tissue (4 µm) were used to determine ZO-1 protein in mice as previously described (Brandt et al. 2019). In brief, deparaffinized sections were treated with protease and incubated with a specific primary ZO-1 antibody (Thermo Fisher Scientific, Waltham, MA, USA) followed by an incubation with secondary antibody linked with peroxidase. Sections were then incubated with diaminobenzidine (Dako Staining Solutions, Agilent Technologies, Vienna, Austria). The extend of staining of 4-HNE protein adducts and ZO-1 was evaluated using a microscope (Leica DM6 B, Leica, Wetzlar, Germany) and analysis software (Leica Applications Suite X, Leica, Wetzlar, Germany) as previously explained (Jin et al. 2015; Brandt et al. 2019).

### RNA isolation, cDNA synthesis and real-time PCR

RNA from livers was isolated with TRItidy G (Applichem, Darmstadt, Germany) as previously detailed (Sánchez et al. 2024). Subsequently, cDNA was synthesized using 400 ng of total RNA following the protocol outlined in the Biozym cDNA Synthesis Kit (Biozym Scientific GmbH, Hessisch Oldendorf, Germany). Following cDNA synthesis, qRT-PCR was conducted using the Biozym Blue S'Green qPCR Kit (Biozym Scientific GmbH, Hessisch Oldendorf, Germany) with primers specifically targeting the following unfolded protein response (UPR) marker genes: glucose-regulated protein 78 (*Grp78* = *Hspa5*), C/EBP homologous protein (*Chop* = *Ddit3*), spliced form of X-box binding protein 1 (*Xbp1s*), the spliced and activated variant of *Xbp1*, indicative of an activated IRE1a-XBP1 pathway. Moreover, lymphocyte antigen 6 family member C1 (*Ly6c1*) expression was detected. Gene expression was normalized to *18S* as a reference gene and quantified using the ΔΔCt method. All primer sequences can be found in Table 1. The primer sequences for the specific detection of Xbp1s were described previously (Yoon et al. 2019).

**Table 1:** Primer sequences

	Forward (5′- 3′)	Reverse (5′- 3′)
*18S*	GTA ACC CGT TGA ACC CCA TT	CCA TCC AAT CGG TAG TAG CG
*Chop*	AGG AGA AGG AGC AGG AGA AC	AGA GAC AGA CAG GAG GTG ATG
*Grp78*	CAC GTC CAA CCC CGA GAA	ATT CCA AGT GCG TCC GAT G
*Ly6c1*	AGA AAG AGC TCA GGG ACT GC	AAA GAA AGG CAC TGA CGG GT
*Xbp1s*	GCT GAG TCC GCA GCA GGT	GCT GAG TCC GCA GCA GGT

*Chop* C/EBP homologous protein, *Grp78* glucose-regulated protein 78, *Ly6c1* lymphocyte antigen 6 family member C1, *Xbp1s* spliced form of X-box binding protein 1

### Microbiota analysis

DNA was extracted from rinsed small intestinal samples with FastDNA^™^ Spin Kit as previously detailed (Kaewtapee et al. 2017). Two rounds of PCR were used to create the sequencing library, targeting V1-V2 regions of the rRNA genes as previously described in detail (Cabrita et al. 2023). Sequencing was performed with the 250 bp paired-end Illumina NovaSeq 6000 platform. Raw fastq files were demultiplexed by Sabre (https://github.com/najoshi/sabre) and then imported to the Qiime2 (Bolyen et al. 2019) for bioinformatic analyses. Primers/adapter sequences were removed by the q2-cutadapt plugin (Martin 2011). Denoising, quality filtering, merging paired reads, and chimeras removal were performed by the q2-dada2 (Callahan et al. 2016) plugin. Resulted amplicon sequence variants (ASV) were annotated with VSEARCH-based consensus (Rognes et al. 2016) and pre-fitted sklearn-based classifiers (Pedregosa et al. 2011) against the Silva database (v138.1, 16S 99%) (Quast et al. 2012). The reference sequences for the taxonomy assignment were obtained and preprocessed by RESCRIPt (Robeson et al. 2021). Alpha diversity was estimated by Shannon’s entropy (Shannon, 1948) and Faith’s phylogenetic diversity (Faith 1992), and beta diversity by the robust Aitchison distances (Martino et al. 2019). Raw sequences were deposited to the European Nucleotide Archive (ENA) under accession number PRJEB73791.

### Statistical analysis

For statistical analysis PRISM (Version 7.03, GraphPad Software, Inc.) was used. Outliers were determined with Grubb´s test and if data were not homogenous in variance, data were log-transformed. In mouse alcohol feeding experiments a two-factorial analysis of variance (ANOVA) was used to determine statistical differences between groups followed by Tukey´s post hoc test whereas a one-factorial ANOVA was used to compare results obtained in the everted sac experiments. Data are displayed as means ± standard error of means (SEM) or as box and whisker plots. *P* < 0.05 was defined to be significant. Statistical analyses of alpha diversity indices were performed by the ANOVA test, and beta diversity distances by the Adonis test (999 permutations). All P-values obtained from multiple comparisons were adjusted using the Benjamini–Hochberg procedure. Genera or features found in all the animals within a given group were defined as “Core features”. A Venn diagram was created with the “Venny4py” package for Python.

## Results

### Effect of supplementing choline on markers of liver damage and inflammation of ethanol-fed female mice

**Fig. 1:**
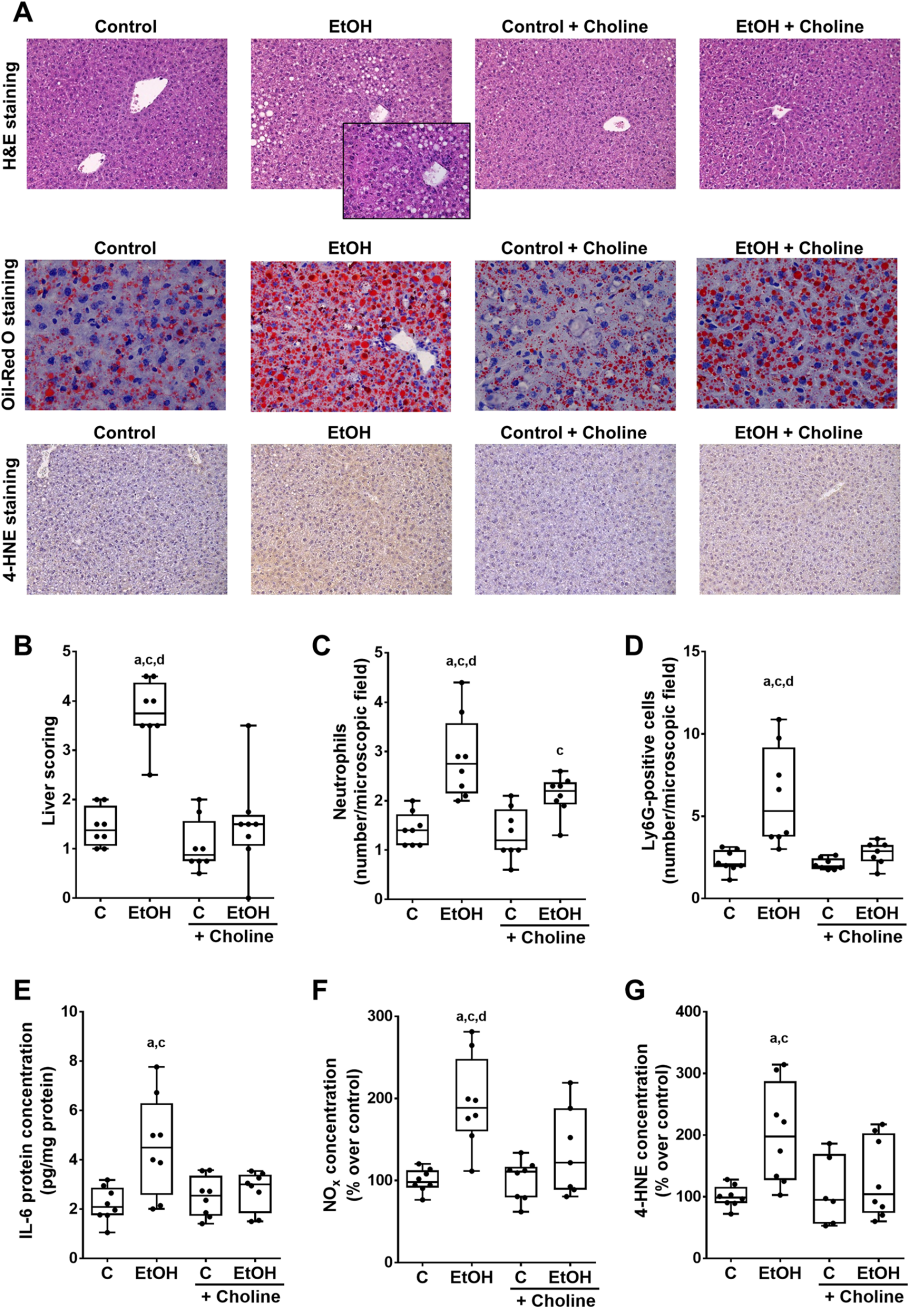
Effect of supplementing choline on markers of liver damage in EtOH-fed female C57BL/6 J mice. Representative pictures of (**A**) hematoxylin & eosin (H&E, magnification 200 × and 400 x) and Oil-Red O staining in liver tissue (magnification 630 x), 4-hydroxynonenal (4-HNE) protein adduct staining (magnification 200 x), (**B**) scoring of liver sections, number of (**C**) neutrophil granulocytes and (**D**) Ly6G-positive cells per microscopic field, (**E**) interleukin 6 (IL-6) protein concentration, (**F**) nitric oxide (NO_x_) concentration in liver tissue as well as (**G**) densitometric analysis of 4-HNE protein adducts. Data are presented as box and whisker plots, n = 8/group except for (**G**) n = 6–8/group. ^a^*p* < 0.05 compared with mice fed the C diet, ^c^*p* < 0.05 compared with mice fed the C + Choline diet, and ^d^*p* < 0.05 compared with mice fed the EtOH + Choline diet. *C* control Lieber DeCarli diet, *EtOH* ethanol-enriched Lieber DeCarli diet, *Ly6G* lymphocyte antigen 6 complex locus G6D

While caloric intake was similar between groups, absolute body weight and body weight gain were significantly lower in mice fed the ethanol-enriched Lieber DeCarli diet supplemented with choline compared to control group (Table 2). Also, alcohol intake was similar between alcohol groups regardless of additional treatments (Table 2). As shown by others before (Hall et al. 2001), the chronic intake of the ethanol containing Lieber DeCarli diet resulted in the development of a mild steatosis and early signs of inflammation (Fig. 1A, B). In contrast, in animals fed the alcohol diet enriched with choline, the development of ALD, as assessed by scoring liver histology, was significantly attenuated (p < 0.05 compared to ethanol-fed mice). Similar results were shown when livers sections were stained with Oil-Red O. Representative pictures of the Oil-red O staining is shown in Fig. 1A. Indeed, while still showing fat accumulation there was almost no macrovesicular fat accumulation visible in livers of ethanol-fed mice treated with choline. Also, inflammation was almost at the level of controls in ethanol-fed mice concomitantly treated with choline. In contrast, ALT activities in plasma were similarly and significantly elevated in both ethanol-fed groups compared to controls (Table 2). Moreover, mRNA levels of the endoplasmic reticulum (ER) stress markers *Grp78*, *Chop* and *Xbp1s* in livers were similar between groups (Table 3). In line with the findings for liver histology, the number of neutrophils and Ly6G-positive as well as F4/80-positive cells in liver tissue was also significantly higher in mice fed the plain ethanol diet compared to all other groups (Fig. 1C, D, Table 2). The number of neutrophils in liver of ethanol-fed mice concomitantly treated with choline was only significantly higher than in mice fed the control diet enriched with choline (Fig. 1C). The number of F4/80- and Ly6G-positive cells in livers of mice fed ethanol and receiving choline were at the levels of controls while mRNA expression of *Ly6c1* was similar between groups (Fig. 1D, Table 2). Moreover, protein levels of IL-6 in liver tissue were significantly higher in ethanol-fed mice compared to both control groups, while not differing between ethanol-fed mice receiving choline and all other groups (Fig. 1E). IL-1β protein levels in livers were significantly higher in ethanol-fed mice compared to control groups and ethanol enriched with choline groups (Table 2). The concentration of NO_x_ in liver tissue was also significantly higher in ethanol-fed mice than in all other groups while not differing between ethanol-fed mice concomitantly treated with choline and controls (Fig. 1F). Also, the concentration of 4-HNE protein adducts in liver tissue was significantly higher in livers of ethanol-fed mice compared to both control groups. Differences alike were not found when comparing 4-HNE protein adduct levels between ethanol-fed mice concomitantly treated with choline and controls (Fig. 1A, G).

**Table 2:** Effect of supplementing choline on caloric intake, body and liver weight as well as liver damage and inflammation in EtOH-fed female C57BL/6 J mice

	C	EtOH	C + Choline	EtOH + Choline
Caloric intake (kcal/g bw)	0.38 ± 0.01	0.35 ± 0.01	0.38 ± 0.01	0.36 ± 0.01
Ethanol intake (g/day)^#^	–	0.36 ± 0.01	–	0.35 ± 0.01
Body weight (g)	23.4 ± 0.7	21.9 ± 0.4	22.9 ± 0.6	20.9 ± 0.4^a^
Absolute body weight gain (g)	4.1 ± 0.5	1.8 ± 0.4^a,c^	3.4 ± 0.3	1.7 ± 0.3^a,c^
Liver weight (g)	1.0 ± 0.04	1.2 ± 0.02^a,c^	1.0 ± 0.02	1.1 ± 0.03
Liver to body weight ratio (%)	4.3 ± 0.1	5.4 ± 0.1^a,c^	4.3 ± 0.0	5.1 ± 0.1^a,c^
ALT (U/L)	17.8 ± 1.5	51.8 ± 6.2^a,c^	15.8 ± 2.3	38.5 ± 5.1^a,c^
F4/80-positive cells (number/microscopic field)	10.5 ± 0.6	28.1 ± 1.8^a,c,d^	9.5 ± 0.5	9.5 ± 0.6
*Ly6c1* mRNA expression (% of control)	100.0 ± 9.3	86.8 ± 7.3	89.7 ± 11.2	127.1 ± 27.2
IL-1β protein (pg/mg protein)	22.0 ± 1.7	42.5 ± 6.7^a,d^	23.7 ± 3.9	22.2 ± 4.0

Data are shown as means ± SEM, n = 7–8/group

^#^Ethanol intake when feeding 5% ethanol-enriched Lieber DeCarli diet

^a^*p* < 0.05 compared with mice fed the C diet

^c^*p* < 0.05 compared with mice fed the C + Choline diet

^d^*p* < 0.05 compared with mice fed the EtOH + Choline diet

*ALT* alanine aminotransferase, *C* control Lieber DeCarli diet, *EtOH* ethanol-enriched Lieber DeCarli diet, *IL* interleukin, *Ly6c1* lymphocyte antigen 6 family member C1

**Table 3:** Effect of supplementing choline on markers of hepatic ER stress in EtOH-fed female C57BL/6 J mice

	C	EtOH	C + Choline	EtOH + Choline
*Grp78* mRNA^#^	1.0 ± 0.1	0.6 ± 0.1	1.2 ± 0.1	1.1 ± 0.2
*Chop* mRNA^#^	1.0 ± 0.1	1.1 ± 0.2	1.2 ± 0.2	1.8 ± 0.2
*Xbp1s* mRNA^#^	1.0 ± 0.3	0.8 ± 0.2	1.4 ± 0.3	1.1 ± 0.2

Data are shown as means ± SEM, n = 5–8/group

^#^relative expression normalized to control

*C* control Lieber DeCarli diet, *Chop* C/EBP homologous protein, *EtOH* ethanol-enriched Lieber DeCarli diet, *Grp78* glucose-regulated protein 78, *Xbp1s* spliced form of X-box binding protein 1

### Effect of supplementing choline on markers of intestinal permeability and NO_x_ concentration in small intestinal tissue of ethanol-fed female mice

**Fig. 2:**
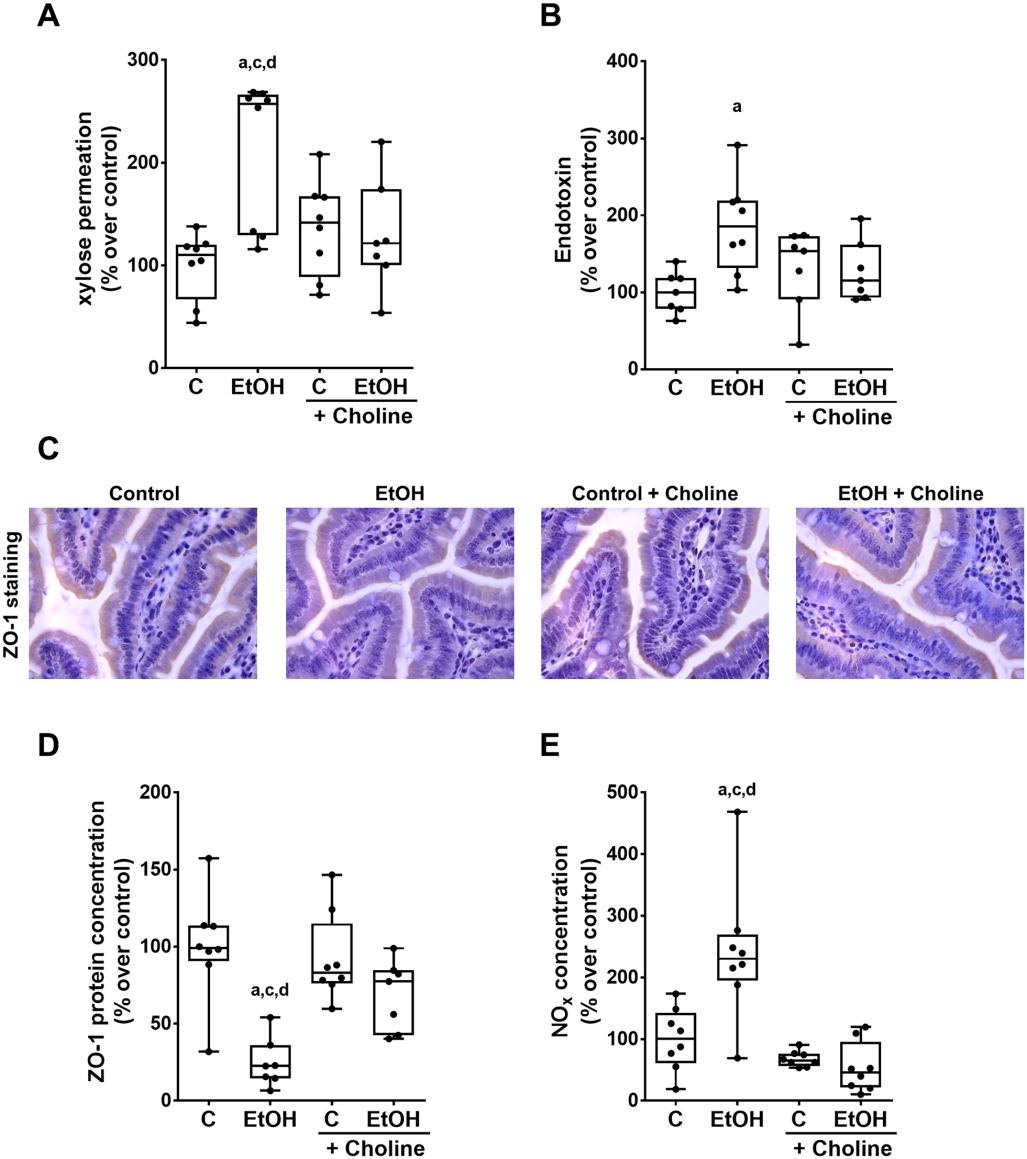
Effect of supplementing choline on markers of intestinal permeability in EtOH-fed female C57BL/6 J mice. (**A**) Xylose permeation assessed in small intestinal everted tissue sacs, (**B**) plasma bacterial endotoxin levels, (**C**) representative pictures (magnification 630 x) and (**D**) densitometric analysis of zonula occludens-1 (ZO-1) protein as well as (**E**) nitric oxide (NO_x_) concentration in small intestinal tissue. Data are presented as box and whisker plots, n = 7–8/group. ^a^*p* < 0.05 compared with mice fed the C diet. ^c^*p* < 0.05 compared with mice fed the C + Choline diet, and ^d^*p* < 0.05 compared with mice fed the EtOH + Choline diet. *C* control Lieber DeCarli diet, *EtOH* ethanol-enriched Lieber DeCarli diet

As acute and chronic alcohol intake has been shown to alter intestinal permeability (Rao 2009) and some studies suggest that choline may be critical in maintaining intestinal barrier function (Arias et al. 2020), we next determined if the protective effects of an oral supplementation of choline on the development of ALD were associated with alterations of intestinal barrier function in the small intestine. Indeed, permeation of xylose as assessed ex vivo in everted small intestinal tissue sacs was significantly higher in mice fed the plain ethanol diet than in all other groups (Fig. 2A). In contrast, xylose permeation was almost at the level of controls in small intestinal tissue of mice fed the ethanol diet enriched with choline (Fig. 2A). Moreover, plasma bacterial endotoxin levels were significantly higher in mice fed the ethanol diet compared to control diet-fed mice. Differences alike were not found between mice fed the ethanol diet enriched with choline and controls (Fig. 2B). Protein levels of the tight junction protein ZO-1 were significantly lower in the small intestinal tissue of mice fed the plain ethanol diet whereas in small intestinal tissue of mice fed the ethanol diet enriched with choline protein levels of ZO-1 were almost at the level of controls (Fig. 2C, D). The protective effects of the oral supplementation of choline on markers of intestinal permeability in ethanol-fed mice were related to the protection of the induction of NO_x_ levels in the small intestine found in mice fed the plain ethanol diet (Fig. 2E). Indeed, in the small intestinal tissue of EtOH + choline-fed mice, NO_x_ concentration was almost at the level of controls while in EtOH-fed mice NO_x_ levels in small intestinal tissue were significantly higher than in all other groups (Fig. 2E).

### Effect of supplementing choline on intestinal microbiota composition in the small intestine of ethanol-fed female mice

**Fig. 3:**
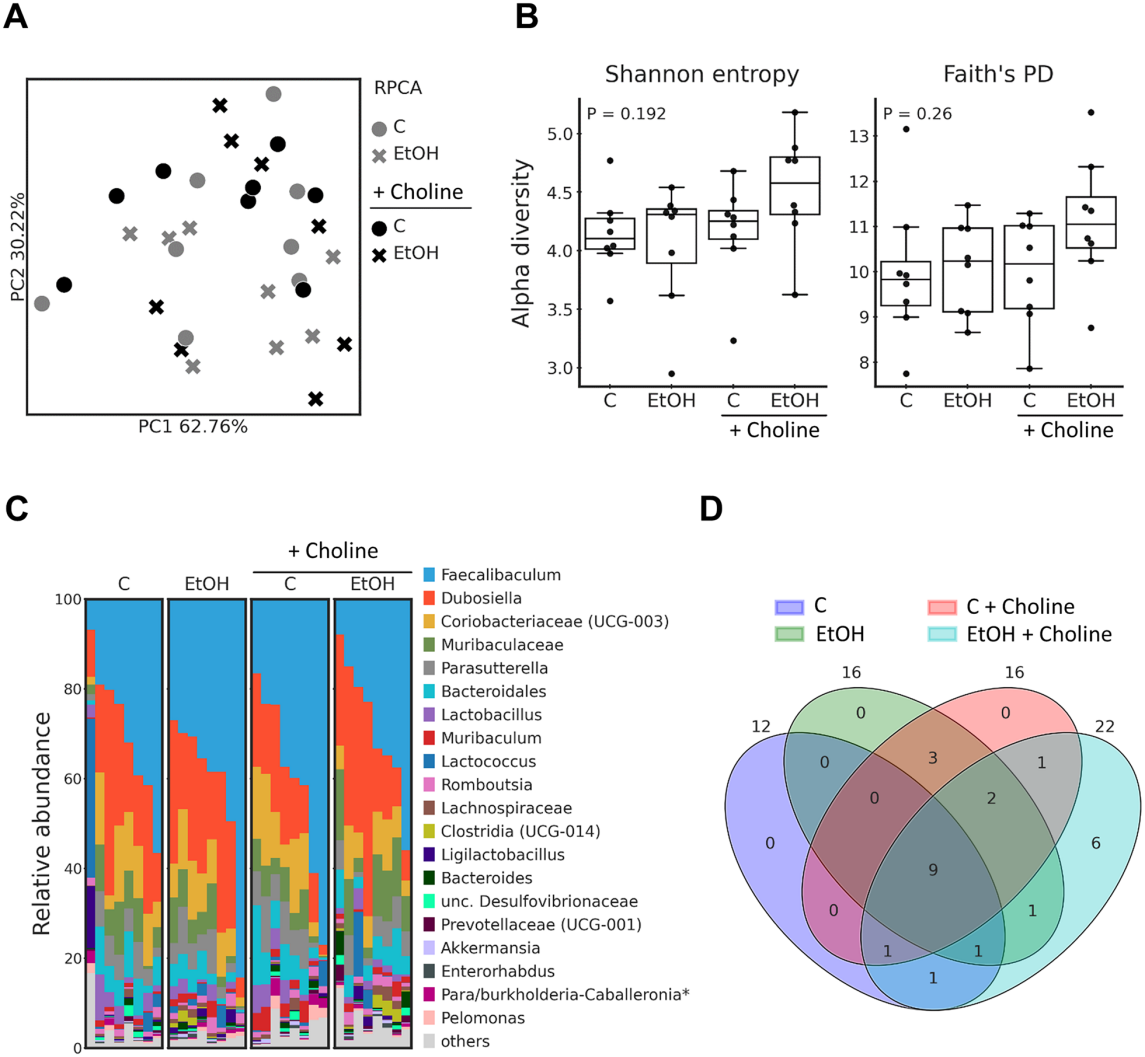
Effect of supplementing choline on microbiota composition in small intestine in EtOH-fed female C57BL/6 J mice. (**A**) PCoA plots based on robust Aitchison distances (RPCA), (**B**) alpha diversity based on Shannon entropy and Faith´s phylogenetic indices, (**C**) relative abundances of bacterial genera (for sequences that were not annoted to the genus level last available level is shown) as well as (**D**) Venn diagram of group core features at genus level. Indices were shown as box plots, n = 8/group. *C* control Lieber DeCarli diet, *EtOH* ethanol-enriched Lieber DeCarli diet

To assess if the protective effects of choline on intestinal barrier function in alcohol-fed mice were associated with changes in intestinal microbiota community structure in the small intestine, 16S rRNA gene sequencing was performed. Neither beta diversity (Fig. 3A) nor alpha diversity based on Shannon entropy or Faith’s phylogenetic diversity indices (Fig. 3B) revealed any significant differences between groups. As shown in Fig. 3C, among all groups and animals, the *Faecalibaculum* genus was the most abundant, followed by *Dubosiella* and classified by the Silva database as “UCG-003” group members of *Coriobacteriaceae* (Fig. 3C). The highest number of core features was found in the ethanol-fed mice concomitantly treated with choline (number of core features: 22), followed by those only fed ethanol (number of core features: 16) and control diet-fed mice treated with choline (number of core features: 16). In mice only fed a control diet the number of core features was 12. Of these, 9 features were shared among all the groups: *Dubosiella*, *Faecalibaculum*, *Lactobacillus*, *Ligilactobacillus*, *Romboutsia*, and unclassified to the genus level members of *Desulfovibrionaceae*, *Bacteroidales*, *Muribaculaceae* and *Coriobacteriaceae* (UCG-003). While control-fed, control + choline-fed and EtOH-fed mice had no features unique to that group, EtOH + choline-fed mice had six (*Enterorhabdus*, *Marvinbryantia*, *Ruminococcus* and unclassified *Prevotellaceae* (UCG-001), *Rhodospirillales* and *Clostridia* (UCG-014)) (Fig. 3D).

### Effect of choline and the choline oxidase inhibitor dimbunol on ethanol-induced intestinal permeability and NO_x_ concentration in everted small intestinal tissue sacs

**Fig. 4:**
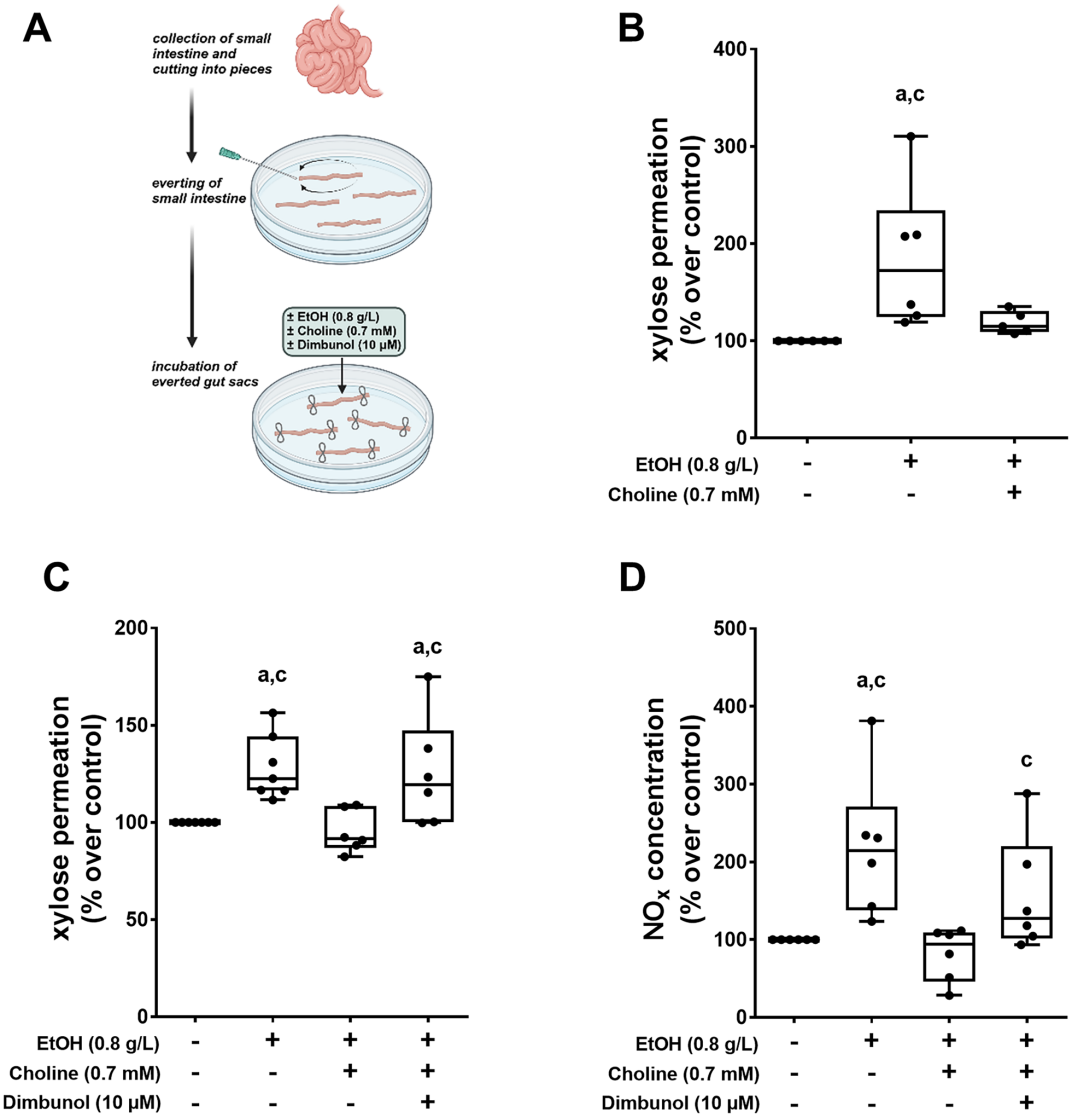
Effect of choline on EtOH-induced intestinal barrier dysfunction in small intestinal everted tissue sacs. (**A**) Schematic drawing of experimental set-up, xylose permeation of everted gut sacs (**B**) treated with EtOH and choline and (**C**) treated with EtOH, choline and choline oxidase inhibitor (dimbunol) as well as (**D**) nitrite (NO_x_) concentration in everted gut sacs treated with EtOH, choline and dimbunol. Data are presented as box and whisker plots, for (**B**) n = 5–6/group, (**C**) n = 6–7/group and (**D**) n = 6/group. ^a^*p* < 0.05 compared with control everted gut sacs, ^c^*p* < 0.05 compared with EtOH + Choline treated everted gut sacs. (**A**) was created with BioRender.com

To further delineate molecular mechanisms underlying the protective effects of an oral supplementation of choline in the development of ALD and the associated intestinal barrier dysfunction in the small intestine, we employed an ex vivo everted small intestinal tissue sac model (Fig. 4A). In line with the findings in vivo, the concomitant treatment of everted tissue sacs with choline attenuated the significant ethanol-induced increase in xylose permeation (Fig. 4B, C) being indicative of intestinal barrier dysfunction and being associated with significantly higher NO_x_ tissue levels (Fig. 4D).

To determine if a lack of the formation of betaine formed from choline through choline oxidase is critical in the development of alcohol-related intestinal barrier dysfunction, small intestinal everted tissue sacs pre-incubated with the choline oxidase inhibitor dimbunol and choline were challenged with ethanol. The protective effects found on xylose permeation and NO_x_ levels in tissue sacs concomitantly treated with choline while being challenged with alcohol were almost completely abolished when the choline oxidase inhibitor dimbunol was present (Fig. 4C, D).

## Discussion

Chronic elevated alcohol intake is among the leading causes of liver damage worldwide (WH Organization 2018). Despite intense research efforts therapeutic options still mainly focus on alcohol abstinence which is often afflicted with a high relapse rate (Batra et al. 2016; Manning et al. 2021). A supplementation of the essential nutrient choline has been suggested before to improve markers of liver damage both in pre-clinical models but even more so in humans with ALD (Mehedint and Zeisel 2013). However, molecular mechanisms underlying the protective effects of an oral supplementation of choline have not yet been clarified. Herein, using a Lieber DeCarli feeding model in female C57BL/6 J mice, we found that an oral supplementation of choline attenuated the development of ALD. Indeed, the oral supplementation of choline markedly attenuated the development of macrovesicular steatosis found in mice only fed ethanol being associated with a protection from the development of early signs of inflammation e.g., infiltration of neutrophils and F4/80-positive cells in the liver and inflammatory foci. Expression of *Ly6c1* mRNA used as a marker of macrophages derived from differentiated monocytes (Teh et al. 2019) was unchanged suggesting that at this stage of ALD, the recruitment of Ly6C-positive cells was not prevalent. Also, the increase in NO_x_ and 4-HNE protein adducts both shown to be indicative of the formation of reactive oxygen species (Zarkovic et al. 2017; Pierini and Bryan 2015) was attenuated in livers of mice treated with choline while ingesting the alcohol diet. It has been shown before by others that an induction of inducible nitric oxide synthase (iNOS) and an elevated formation of reactive oxygen species is critical in the development of ALD and that attenuating the induction of iNOS or formation of reactive oxygen species through interfering with the NADPH oxidase is associated with a protection from the development of ALD in rodents (McKim et al. 2003). Furthermore, protein levels of IL-1β and IL-6 were also almost at the level of controls of choline-treated mice fed alcohol. Both serum and hepatic IL-6 levels but also IL-1β levels have been shown before to be elevated in patients with ALD and animal models of ALD suggesting that IL-6 expression is related to disease development and severity (Hill et al. 1992; Horiguchi et al. 2008; Cui et al. 2015). However, as reviewed in detail by Gao et al. (2012), it remains to be determined if the induction of IL-6 is part of the host defense strategy especially as IL-6 deficiency in mice may increase susceptibility to alcohol-induced liver damage (El-Assal et al. 2004). Somewhat contrasting these findings, liver to body weight ratios and ALT activity in plasma were similar between alcohol-fed groups. However, data varied considerably in the ethanol groups. Also, it has been suggested before that ALT may be more indicative of hepatic fat accumulation than inflammation (Moriles et al. 2020) which was changed to a lesser extend in the present study compared to the effects found on inflammation. Furthermore, markers of ER stress reported before to be induced in settings of ALD (Na et al. 2023) were unchanged in both ethanol-fed groups. While contrasting the findings of others in more severe stages of ALD, in which ALD was induced by other measures e.g., Tsukamoto French model (Ji et al. 2005), the results of our study are in line with the findings of others employing the Lieber DeCarli diet (Galligan et al. 2012). However, there are also contradictory reports showing that markers of ER stress may be induced in liver tissue of mice fed an ethanol containing Lieber DeCarli diet (Fernandez et al. 2013). Recently, it has been discussed that ER stress may occur constitutively in or secondary to the state of ALD further suggesting that in the present study where mice only showed early signs of ALD, markers of ER stress may not yet have been induced.

Taken together, our results further bolster the findings of others reporting that supplementing choline may bear protective effects on the development of early stages of ALD and that these may be related to a protection against not only fat accumulation but also inflammation. However, our data by no means preclude that when taken in higher doses or over an extended period of time like shown in older studies in baboons (Lieber et al. 1994) choline may actually exert adverse and even toxic effects on the liver.

### How does an oral choline supplementation attenuate the development of ALD?

Results of several studies suggest that not only the hypermetabolic state in the liver resulting from alcohol metabolism and the associated shift in the ratio of NADH^+^/H^+^ and NAD^+^ may be critical in the development of ALD but rather, alterations of intestinal microbiota composition and impairments of intestinal barrier function may also be critical herein (for overview see (Schöler and Schnabl 2024)). Indeed, it has been shown that the development of ALD is closely related to an increased permeation of PAMPs, and herein, especially of bacterial endotoxin (Rao 2009). Improving intestinal barrier function or interrupting TLR(4) signaling in liver tissue is related with a protection from the development of ALD (Rao 2009). In the present study, the protective effects of the oral supplementation of choline were associated to a protection from the increase in small intestinal permeability as determined by xylose permeation in small intestinal tissue and bacterial endotoxin levels in portal blood as well as assessing the loss of tight junction proteins. In the present study, bacterial endotoxin levels were significantly higher in ethanol diet-fed mice compared to controls while similar differences were not found in ethanol-fed mice concomitantly treated with choline. Results of more recent studies employing animal and cell culture models also suggest that the attenuation of the loss of tight junction proteins in settings of acute and chronic alcohol exposure may be related to altering the bioavailability of betaine and attenuating the induction of NO (Wu et al. 2020; Tang et al. 2009). Also attenuating the overproduction of NO with iNOS inhibitors in small intestinal tissue has been shown to be related to a marked decrease of bacterial endotoxin levels in serum of rats fed ethanol (Tang et al. 2009). In line with these findings, in the present study, NO_x_ levels in small intestinal tissue of ethanol-fed animals concomitantly treated with choline were almost at the level of controls. Furthermore, the protective effects of choline on ethanol-induced intestinal permeability but also the formation of NO_x_ in small intestinal tissue were almost completely attenuated in everted small intestinal tissue sacs treated with the choline oxidase inhibitor dimbunol further supporting the hypothesis that an altered formation of betaine in small intestine may be critical in the development of alcohol-related intestinal barrier dysfunction. However, if betaine levels in small intestinal tissue were altered in alcohol-fed mice and if the supplementation of choline abolished these alterations remains to be determined.

Interestingly and contrasting the findings of others in feces and a limited number of studies in the upper gastrointestinal tract showed marked differences in both human and rodents between non-drinkers and alcohol-drinkers with and without ALD with respect to microbiota composition (Schöler and Schnabl 2024; Wang et al. 2016; Day and Kumamoto 2022), in the present study, microbiota composition in the small intestine seemed widely unaffected by the addition of ethanol or ethanol and choline to the diet. Differences between the present study and those of others might have been related to differences in diet and the amount of feeding (here: Lieber DeCarli diet vs. others: Tsukamoto & French models) as well as the stage of disease and species. Also, while there were no overt differences in small intestinal microbiota composition it cannot be ruled out that metabolism of bacteria was altered. Indeed, six core genera were listed as core genera in ethanol-fed mice concomitantly fed choline while not being listed in all other groups. Still, the results suggest that the protective effects of choline on the development of ALD may not have resulted from its effect on intestinal microbiota composition in the small intestine.

In summary, our results suggest that an oral supplementation of choline at least in part attenuated the development of intestinal barrier dysfunction through altering intestinal choline metabolism thereby probably increasing bioavailability of betaine in small intestine. However, further studies are needed to determine molecular measures by which choline and betaine interfere with NO synthesis, and subsequently, the loss of tight junction proteins. Also, it remains to be determined if choline has similar effects in humans. Moreover, our results do not preclude that choline may also directly affect liver metabolism but rather suggest that one of the mechanisms of action underlying the beneficial effects of an oral choline supplementation in settings of ALD is related to its effect on small intestinal mucosa.

## Conclusion

Taken together, the results of the present study add further weight to previous findings in settings of diet-induced metabolic associated steatohepatitis (Al Rajabi et al. 2014) that enriching the diet with choline may diminish the development of ALD. Our data also suggest that herein the effects of choline on intestinal barrier function may be critical and that choline may attenuate the development of alcohol-induced intestinal barrier dysfunction by mechanisms involving a decreased formation of NO in small intestinal mucosa. Further studies are needed to determine (1) if an oral choline supplementation also has curative effects in human especially when later stages of ALD e.g., fibrosis are prevalent, and (2) if an oral supplementation of choline also prevents intestinal barrier dysfunction in patients with ALD. Also, it needs to be kept in mind that even if an oral choline supplementation attenuates the development of ALD, it may not attenuate the effects of alcohol on other organs including cognitive impairments associated with the acute alcohol intake.

## Data Availability

Data can be made available upon reasonable request.

## Abbreviations

4-HNE: 4-Hydroxynonenal protein adducts
ALD: Alcohol-related liver disease
ALT: Alanine aminotransferase
C: Control Lieber DeCarli diet
Chop: C/EBP homologous protein
ER: Endoplasmic reticulum
EtOH: Ethanol-enriched Lieber DeCarli diet
Grp78: Glucose-regulated protein 78
IL: Interleukin
iNOS: Inducible nitric oxide synthase
Ly6c1: Lymphocyte antigen 6 family member C1
Ly6G: Lymphocyte antigen 6 complex locus G6D
NO: Nitric oxide
NO_x_: Nitrite
PAMPs: Pathogen-associated molecular pattern
TLR: Toll-like receptor
Xbp1s: Spliced form of X-box binding protein 1
ZO-1: Zonula occludens 1
